# Chicken liver and eggshell crackers are a safe and affordable animal source food for overcoming micronutrient deficits during pregnancy and lactation in Indonesia: a double-blind, cluster randomized controlled trial (SISTIK Growth Study)

**DOI:** 10.1186/s12884-025-07899-0

**Published:** 2025-08-06

**Authors:** Aly Diana, Sofa Rahmannia, Yenni Zuhairini Suhadi, Dimas Erlangga Luftimas, Haidar Rizqi, Afini Dwi Purnamasari, Ayunda Jihadillah, Mohammad Brachim Ansari, Dearly Ayu Zahrotun Haq, Aisyah Nur Pratiwi, Dina Novtyana Puspita, Yeni Intan Kusuma Dewi Affandy, Christopher M. A. Frampton, Umi Fahmida, Lisa A. Houghton, Rosalind S. Gibson

**Affiliations:** 1 Department of Public Health, Faculty of Medicine, Universitas Padjadjaran, Bandung, 40161 Indonesia; 2 Southeast Asian Ministers of Education Organization Regional Center for Food and Nutrition (SEAMEO RECFON), Jakarta, 13120 Indonesia; 3 Faculty of Medicine, Universitas Pasundan, Bandung, 40117 Indonesia; 4 School of Population and Global Health, University of Western Australia, Crawley, 6009 Australia; 5 Nutrition Working Group, Faculty of Medicine, Universitas Padjadjaran, Bandung, 40161 Indonesia; 6 Department of Medicine, University of Otago, Christchurch, 8014 New Zealand; 7 Department of Nutrition, Faculty of Medicine, Universitas Indonesia—Dr. Cipto Mangunkusumo General Hospital, Jakarta, 10430 Indonesia; 8 Department of Human Nutrition, University of Otago, Dunedin, 9016 New Zealand

**Keywords:** Anaemia, Birth length, Birth weight, Fortified wheat flour, Indonesia, Lactation, Length velocity, Linear growth, Micronutrient enriched crackers, Pregnancy

## Abstract

**Background:**

Poor diets and micronutrient deficiencies during pregnancy and lactation are common in Indonesia, potentially affecting linear growth. National maternal programs focus predominantly on iron-folic acid (IFA) supplementation, but adherence is poor. We explored a strategy utilizing locally available micronutrient-rich foods in the form of micronutrient-enriched crackers (MEC) to improve neonatal and infant growth.

**Methods:**

The Sustainable Intervention of Supplementation to Improve Kid’s (SISTIK) Growth Study was a double-blind, cluster randomized controlled trial conducted in 28 village clusters in Sumedang, Indonesia (Nov 2020–Jan 2023). Villages—unit of randomization—were stratified by size and assigned to receive either MEC or standard wheat crackers (SWC), along with nutrition and health education, morbidity control, and national IFA supplementation programs. Eligible pregnant women (19–35 years; 7–13 weeks gestation) were enrolled participated. The intervention lasted through five months postpartum. Individual mother-infant dyads were the unit of analysis. Primary outcomes were birth length (cm), attained length (cm), LAZ-score, and length velocity (cm/month) from birth to five months postpartum. Secondary outcomes included birth weight, morbidity, and maternal hemoglobin (Hb) (g/dL). A general linear model, including village as a random effect and intervention as a fixed effect was used.

**Results:**

In each group 137 mother-infant dyads completed the study. Baseline characteristics were balanced. Intervention adherence was low (72–103 gr/week) compared to 525 gr/week (75 gr/day) recommended, with no significant intervention effect on growth outcomes, after adjusting for maternal height, sex, and human milk intake at 5 months. There were no differences between MEC and SWC groups in maternal Hb (mean ± SD) (12.0 ± 1.1 vs. 12.3 ± 1.2 g/dL) and anemia (11.9 vs 11.3%) at 35–36 weeks GA or morbidity. Compared with the same setting earlier at 5 mos pp, mean ± SD LAZ-score were less negative and stunting prevalence was lower in both groups. Overall, across the SISTIK study population, mean ± SD LAZ-score improved from -0.93 ± 0.85 to -0.51 ± 0.91, and stunting prevalence declined from 11 to 6%.

**Conclusions:**

Although this study did not show a treatment effect on the outcomes measured, future research should explore whether providing comprehensive nutrition and health support during pregnancy and post-partum could help reduce stunting in Indonesia.

**Trial registration:**

NCT04564222 (ClinicalTrials.gov); 25/09/2020.

## Introduction

In Southeast Asia, multiple micronutrient deficiencies often coexist among women of reproductive age and remain a public health challenge [1]. In Indonesia, micronutrient deficits in diets and biomarkers have been identified during both pregnancy [2, 3] and lactation [4, 5] with the potential to impair fetal and early post-natal infant growth. Current national intervention programs during pregnancy in Indonesia focus almost exclusively on iron folic acid (IFA) supplementation [3, 6] even though antenatal multiple micronutrient supplements are now recommended in low and middle-income countries by World Health Organization [7] in view of their reported benefits compared with IFA supplement alone. Such benefits include increases in birth weight, and reductions in risk for LBW, preterm birth, or SGA infants [8, 9], although few studies have shown a positive effect on birth length. However, in Indonesia adherence to IFA supplements is poor with only ~ 38% of pregnant women consume at least 90 tablets during pregnancy) [10], raising concerns over the potential compliance to multi-micronutrient supplements. As a result, alternative strategies based on locally available micronutrient-rich foods have been explored [11, 12]. Such an approach can alleviate co-existing multiple micronutrient deficiencies simultaneously without any risk of antagonistic interactions. It can also be adapted to local conditions so that it is acceptable and affordable, providing a more sustainable solution than supplementation. In addition, a food-based approach has the advantage of being community-based, so it can be used to enhance community awareness of micronutrient malnutrition in the future [13].

In this study we use a food-based strategy based on chicken liver and eggshell powder, a combination with the potential to alleviate micronutrients deficits identified earlier among pregnant and lactating women in Indonesia [3–5, 14]. Chicken liver is a readily available, affordable, acceptable, and safe animal-source food in Indonesia, which has been recommended to enhance dietary adequacy for infant and young child feeding [12], and pregnant and lactating women in Indonesia [3, 4]. However, in rural settings, chicken liver has a limited shelf-life. Consequently, to extend the shelf-life, we chose crackers, a common snack in Indonesia as our food vehicle for enrichment. Powdered eggshells were included as an ingredient of the MEC as they are a rich source of bioavailable calcium [15], which was identified earlier as a major deficit characterizing the diets of lactating women residing in the rural setting of this study [4, 5, 14]. Powdered eggshells also contain an insulin-like growth factor (IGF-1), reported to promote fetal and early infant linear growth [16], the primary outcomes of this study. We used technology that was readily transferable through a local industry partner to ensure our micronutrient enriched crackers (MEC) could be produced sustainably, required no cold storage, and were available locally in our resource-poor study setting.

Here we describe a double-blind, cluster randomized controlled trial (RCT) of the effectiveness of our locally produced MEC crackers for neonatal and early infant growth. A cluster design was chosen to minimize contamination between groups due to potential food sharing within communities**.** We applied a shortened dose-to-mother (DTM) deuterium oxide protocol [17, 18] to distinguish between those infants who were truly exclusively and partially breast-fed, and to measure their human milk intake (gr/day) at 5 mos post-partum. In many earlier interventions, uncertainties have existed about whether infants were truly exclusively breastfeeding during early infancy [19], in part because exclusivity is frequently based on maternal or caregiver self-reports known to be subject to self-reporting bias [20].

Many other factors besides dietary inadequacy can impact newborn and infant growth in low-resource households. Of these, anemia in pregnancy, reportedly 48.9% nationally in Indonesia [10], is associated with low birthweight (LBW) and preterm birth, both known to increase risk of subsequent linear growth faltering [21]. Growth faltering is also exacerbated by morbidity as well as the multiple adverse effects of asymptomatic environmental enteric dysfunction (EED) [22, 23]. EED often originates from exposure to fecal contamination due to poor quality of water, sanitation, and hygiene (WASH), a factor identified earlier in our study setting limiting infant linear growth [19]. Consequently, here we have also incorporated maternal strategies focused on WASH and tested household drinking water samples for coliform bacteria [24].

Therefore, the goal of our study was to assess the effectiveness of daily consumption of locally produced MEC by pregnant women at two-time intervals: (i) from 7–13 weeks GA to delivery on newborn length, and (ii) from 7–13 weeks gestation to 5 months post-partum on attained linear growth and linear growth velocity of term breastfed infants. Secondary outcomes included newborn weight, maternal hemoglobin, and maternal and infant morbidity. We chose the primary outcomes in view of the importance of the prenatal environment on subsequent linear growth in low resource households in Indonesia [25], uncertainty about the contribution of intrauterine growth retardation (IUGR) on subsequent linear growth failure, and the government priority to combat the persistent stunting in our study setting [26].

## Methods

### Study design, participants, and enrolment

This Sustainable Intervention of Supplementation to Improve Kid's (SISTIK) Growth Study trial is registered as NCT04564222 (ClinicalTrials.gov) with a double-blind, cluster randomized controlled design. The full trial protocol has been published earlier [27]. The trial was conducted between November 2020 to January 2023 in three subdistricts of Sumedang district, West Java, Indonesia. Sumedang district has a population of 1.1 million and an area of ~ 152 hectares, of which 22% is used for rice paddy plantations. The climate is tropical with rain most months, and only a short dry season. Maternal diets are based on starchy staples (mainly rice); dietary quality is poor with a high risk of micronutrient inadequacies [4]. The most pronounced dietary inadequacies were observed for calcium, vitamin B6, niacin, riboflavin, and vitamin A, with prevalence of inadequacy often exceeding 60–90%. Iron and B12 inadequacy affected approximately half of the mothers, while zinc inadequacy remained comparatively low [4, 5, 14]. The rate of stunting in Sumedang district is higher compared to the national average (i.e., 27.6% vs. 21.6%) [28].

To prepare for the trial, the purpose and methods were explained to district midwives and community health worker (cadres) in three sub-districts of Sumedang district. Next, eligible pregnant women were identified by midwives through village pregnancy registrars. Pregnancy was established initially by retrospective maternal recall of first day of the last menstrual period (LMP), and subsequently confirmed after enrolment by urine test [8]. Participation in the trial was voluntary and women were free to withdraw at any time. Women were eligible if they were generally healthy and: 1) aged 19–35 years; 2) had a gestational age (GA) 7–13 weeks; and 3) were permanent residents. Women with any health concerns during past pregnancies were excluded; see the protocol for exclusion details [27].

### Randomization

A statistician (JJH) in New Zealand, who was not involved in enrolment and data collection, performed the randomization sequence. Trial participants were recruited from 28 clusters (villages) located in the three sub-districts in Sumedang district. Clusters were randomly assigned to either intervention (*n* = 14 villages) or placebo (*n* = 14 villages), with villages of < 100 and ≥ 100 infants stratified using block sizes of 2 and 4, respectively. The randomized sequence was created by Stata 16.1 (StataCorp, College Station, TX).

### Interventions and adherence

The micronutrient-enriched crackers (MEC) were based on chicken liver and powdered eggshells and designed to meet the minimum prevalence of adequacy for eight micronutrients (calcium, iron, zinc, vitamin A, niacin, folate, thiamin, riboflavin) [27] when consumed at a daily dose of 75 gr per day [4]. Nationally fortified wheat flour was the primary ingredient of the SWC, with the addition of *Pangium edule* seeds to mimic the color of MEC. Both MEC and SWC also contained tapioca flour, egg, salt, mushroom bouillon, margarine, and seasoning. Random batches of the products prepared at baseline, midline, and endline were analyzed for macronutrients by proximate analysis and micronutrients by Saraswati Indo Genetech Laboratory (Bogor, Indonesia) (Table 1). For more details of product development and testing, see the protocol [27].

**Table 1:** Average nutritional content of micronutrient-enriched cracker (MEC) and standard wheat crackers (SWC) at baseline, midline, and endline (per 75 g), and contamination assessment

Parameter (average)	MEC (75 g)	SWC (75 g)
Macronutrient^a^		
Protein (%)	9.6	5.4
Fat (%)	24.1	25.7
Carbohydrate (%)	39.5	43.6
Cholesterol (mg)	56.2	11.9
Total energy (kcal)	420.5	432.2
Micronutrient		
Vitamin A Retinol (µg RAE)^a^	161.3	66.1
Thiamine (mg)^b^	0.10	0.07
Riboflavin (mg)^b^	0.43	0.37
Niacin (mg)^b^	1.5	1.0
Vitamin B6 (mg)^b^	0.93	0.07
Folate (µg)^b^	49.6	44.6
Vitamin B12 (µg)^b^	0.77	0.07
Calcium (mg)^a^	506.0	74.9
Iron (mg)^a^	4.0	4.2
Zinc (mg)^a^	2.7	2.2
Sodium (mg)^a^	299.7	406.7
Chemical and biological contamination analysis^a^		
Pb	Not detected	Not detected
Hg	Not detected	Not detected
Cd	Not detected	Not detected
As	Not detected	Not detected
Sn	Not detected	Not detected
*Salmonella sp.*	Negative	Negative
*Bacillus cereus*	< 10	< 10
*Enterobacteriaceae*	0	0
*Coagulase positive staphylococci*	< 10	< 10

Wheat flour was fortified following the mandatory policy from the Indonesian government with thiamin (2.5 mg/kg), riboflavin (4 mg/kg), iron (50 mg/kg), zinc (30 mg/kg), and folic acid (2 mg/kg) (Menteri Kesehatan Republik Indonesia. Keputusan Menteri Kesehatan Republik Indonesia nomor 1452/Menkes/SK/X/2003 tentang Fortifikasi Tepung Terigu. Jakarta; 2003)

^a^Laboratory analysis at baseline, midline, and endline

^b^Calculated based on recipe at baseline; laboratory analysis at midline and endline

The acceptability of the MEC was assessed in a single-blinded, two-phase acceptability trial involving 81 non-pregnant women aged 19–35 years in Ujung Berung Sub-district, Bandung City [29]. Briefly, in phase 1, participants sampled both MEC and SWC in a test feeding session and rated each product using a 7-point cued facial response scale, evaluating colour, smell, flavour, and texture. MEC received favorable ratings, with no significant differences (*p*-value > 0.05) compared to SWC in liking scores for each attribute tested. In Phase 2, participants were randomly assigned to receive a 14-day home supply (75 g/day) of either MEC (*n* = 41) or SWC (*n* = 40). Adherence, determined by weighing unconsumed products, was comparable between groups, with average daily consumption over 14 days of 50.8 ± 23.0 g in the MEC group and 51.0 ± 20.0 g in the SWC group (mean difference: −0.2 g; 95% CI: −6.5 to 6.1; *p* = 0.802).

The products of both treatment groups were tested for their shelf-life (i.e., I year) (Food Technology Laboratory, Universitas Pasundan, Bandung, Indonesia), and for excessive intake of vitamin A (i.e., above Upper Tolerable Level (UL)) (Table 1), risk of exposure to heavy metals, and microbial contamination (Saraswanti Indo Genetech Laboratory, Bogor, Indonesia); all batches analyzed contained less than the UL; see Diana et al. [27] for more details.

All crackers were packaged in identical food grade aluminum foil and labelled with a code (A or B) known only by the manufacturing production manager who had no connection to the study personnel. Study staff, the statistician, and participants were unaware of the product allocation, which was not revealed until the analyses of the primary outcome was completed. Study staff only knew group assignment by the prescribed letter (A or B). The effectiveness of these blinding restrictions was not evaluated.

Home visits were made weekly by trained research assistants (RAs) or village health workers (cadres) to deliver the products which were indistinguishable in color, texture, and packaging, except for the coding (A or B). To avoid an increase in energy intake, all participants were encouraged to consume daily 75 gr of “study” crackers as a replacement for their usual crackers, with a choice of an “original” or “spicy” flavored study cracker to enhance adherence. Each stick weighed approximately 2 g, so participants were expected to consume around 35 to 40 sticks (1.5 handfuls) per day. To avoid any sharing among other family members, participants were given a family cracker package (i.e., 150 gr of MEC or SWC) initially, and then weekly during the study on request. At each home visit, RAs or cadres collected any remaining products, which were weighed using dietary scales (accurate to ± 1 gr) (Kitchen Scale EK3650/EK3651, Camry Electronic Ltd, Guangdong, China) and their weight recorded (in gr/week). Participants with low adherence to the products allocated were supplied with the alternative flavored product during these home visits in an effort to enhance compliance. The mean weight of crackers consumed overall, and adherence rates (gr/week) were examined. Respondents with missing adherence data that accounted for > 60% of the total number of weeks of consumption, were excluded from the adherence calculations (*n* = 2, pregnancy; *n* = 5, lactation).

Analyzed total energy content of the MEC and SWC was similar (~ 425 kcal per 75 gr), whereas the content of protein, vitamin A (as retinol equivalents), B-12, folic acid, and calcium was higher for the MEC than for the SWC (Table 1). The MEC was formulated to be affordable: the price per serving (~ 75 g) was approximately equivalent to the price of two medium-sized chicken eggs in local markets at the time of the study. Wheat flour, fortified at the national level, contributed 66% of the micronutrient content of the SWC.

### Outcomes

#### Measurement of anthropometric status

Primary outcomes were birth length (cm) (for all singleton livebirth infants) and LAZ-score within 24 h of delivery, as well as attained length (cm), length velocity (cm/mos), and LAZ-score [30] at 5 mos post-partum. Other growth outcomes were weight at birth (for all singleton livebirth infants) and at 5 mos post-partum, their respective z-scores, and weight-for-length z-scores [30]. Only z-scores for term infants were reported at birth. In addition, the proportion of infants with LBW (< 2500 g), IUGR (i.e., birth weight < 10th percentile [31], preterm birth and those with z-scores < −1 and < −2 at birth and 5 mos post-partum [30], were also calculated. For IUGR calculation, only infants born between 33 and 42 weeks GA were included [31].

Maternal height and weight were measured at baseline (i.e., 7–13 weeks GA), and used to classify overweight and obesity based on the Asian classification [32]. Weight was measured again at 35–36 weeks gestation, and pregnancy weight gain was calculated based on body mass index (BMI) at 7–13 weeks GA and compared to the recommendations by the U.S National Research Council [33]. Briefly, all anthropometric measurements were conducted by trained, experienced research assistants using calibrated equipment and standardized measurement protocols [27]. Technical error of measurement (TEM), both inter- and intra-examiner, was calculated for each anthropometric measure based on a subsample of 20 infants. All measurements were taken twice, with a third measurement performed if the difference between the first and second exceeded the maximum allowable difference, as defined in the protocol for the WHO international growth standards [34].

#### Measurement of human milk volume (gr/d) and breast-feeding status

Preterm, LBW, and those infants not breastfed were excluded from these variables. Human milk intake was measured via DTM technique at 5 mos post-partum over 14 days. Briefly, pre-dose saliva samples were collected on day 0 from mother-infant dyads, after which mothers received a measured oral dose of diluted deuterium oxide [14]. Next, post-dose saliva samples were collected from mother-infant dyads on days 2 or 3; 8 or 9; and day 14; see Liu et al. (2019) [35] for more details. Following analysis of deuterium enrichment by Fourier transform infrared spectrometry (Agilent 4500), Agilent Technologies, Dansbury, USA), we used a method modified from International Atomic Energy Agency [36] to calculate both the average human milk intake over a 14-d period and to distinguish exclusively and partially breastfed infants at 5 mos post-partum. Breastfeeding classification was based on the infant’s intake of water from sources other than human milk [18, 35–37].

#### Assessment of socio-demographic, health status, and hemoglobin (Hb) status

Information on socio-demographic status was recorded at enrolment by trained RAs using a structured questionnaire. During home visits at enrolment, 35–36 weeks gestation, 2 and 5 mos post-partum, a questionnaire alongside direct observations, when feasible, were also used to record household water and sanitation facilities. Operational definitions by WHO and UNICEF (2012) [38] and Indonesian DHS (2017) [39] were applied to classify household water and sanitation facilities as “improved”. Information on boiling water at point of use was recorded based on maternal self-reports. Records of the maternal use of daily vitamin and/or mineral supplements (with brand names) were collected weekly during home visits. Household water samples at source and drinking water at the point of use were collected at 5 mos post-partum, analyzed for *E.coli* and other coliform bacteria, and later compared with Indonesian Health Ministry criteria [24, 40, 41].

All trial participants also received nutrition and health education provided by the research team at two time points: at baseline (early pregnancy) and after delivery. Topics included: (i) importance of the first 1000 days of life for optimal growth, health, and development [42]; (ii) balanced nutrition during pregnancy and lactation [43]; (iii) WASH, including five key messages for making food consumed safe [44, 45]: and (iv) recommended breastfeeding practices [46]. Leaflets covering these topics were distributed, and participants were given the opportunity to ask questions.

Participants recorded illnesses over 30 days on morbidity calendars at 7–13 weeks, 35–36 weeks, and 2 and 5 mos post-partum, and at 2 and 5 mos for their infants. Specific symptoms recorded for mother-infant dyads were respiratory-related illnesses (runny nose, cough, and sore throat), fever, skin rash, other illnesses, and diarrhea-related illnesses (diarrhea, vomiting, nausea, and stomach pain) with or without fever. Diarrhea was defined as ≥ 3 liquid or semiliquid stools in 24 h. Any mother-infant dyads experiencing illness were referred to the primary health centers. Pregnancy-related symptoms, including seizure and vaginal bleeding were also recorded by the women.

Maternal hemoglobin (Hb) was measured in finger-prick blood by Hemocue 201 + (HemoCue AB, Angelholm, Sweden) for initial screening at 7–13 weeks GA, and in venous blood at both 35–36 weeks GA and 5 mos pp by an automated counter (Sysmex XN 1000, Sysmex Corporation, Kobe, Japan). Maternal anemia was defined as Hb concentration < 3rd percentile at 35–36 weeks gestation [47] and as Hb < 11.0 gr/dL [48].

### Adverse events and safety monitoring

All adverse events, including hospitalizations, adverse pregnancy outcomes and neonatal events were monitored continuously by the Clinical Monitor, recorded as per protocol [27], and reviewed annually by the Data Safety Monitoring Board (DSMB). No interim analysis was deemed necessary. Reasons for all withdrawals were sought and recorded, and follow-ups conducted for any unresolved adverse events. Maternal hypercholesterolemia at third trimester (serum cholesterol > 240 mg/dL) [49] and elevated serum uric acid (> 6 mg/d) [50] were also monitored at 35–36 weeks gestation from finger-prick blood samples.

### Ethics and health protocols for COVID-19 prevention

The trial protocol conforms to the principles outlined in the Declaration of Helsinki 2013 [51], and was approved by Indonesian Health Research Ethics Committee, National Institute of Health Research and Development (HREC-NIHRD), reference number LB.02.01/2/KE.496/2020 and LB.02.01/2/KE.503/2021; protocol version 1.4 (3rd August 2021). Participants gave written informed consent. Project staff referred any ill participants to local medical services and followed up any subsequent treatment. All participants had access through pregnancy and 5 mos post-partum to supplemental iron and folic acid via the national intervention program [52], as well as multiple micronutrients and calcium supplements.

To prevent risk of COVID-19 infection, all project staff and participants complied with the regulations set by the Ministry of Health, Republic of Indonesia [53]. All field assistants involved in data collection were equipped with Personal Protective Equipment (PPE); contact tracing was followed for any participants and/or assistants positive for COVID-19, followed by home self-isolation when necessary.

As a result of the risk of COVID-19 infections and subsequent “lockdown”, there were two types of protocol violations. During March-August 2021, anthropometric measurements for 78 newborns were taken by midwives or by hospital healthcare personnel instead of our trained RAs who were not permitted to make hospital visits. In addition, some follow-up assessments at enrolment (7–13 weeks), 35–36 weeks GA, and 2 and 5 mos post-partum took place beyond the protocol specifications.

### Sample size

For the primary outcome from the second trimester of pregnancy (8–14 weeks) to delivery, a minimum total sample size of 182 (91 in each group) was estimated to detect a 1 cm difference in birth length between MEC and SWC as statistically significant (two-tailed α = 0.05) with 90% power. A 1 cm difference equates approximately to 0.5 SD from WHO Growth Chart) [30]. This calculation incorporated a design effect due to village clusters of 1.1 based on an earlier study in the same setting. A total sample size of approximately 102 in each group would also ensure 90% power to detect a 0.7 cm/month difference in growth velocity from the second trimester of pregnancy to 5 months post-partum. To allow for exclusions due to stillborn or premature birth (~ 10%), failure to breastfeed (~ 15%), and anticipated attrition over the trial duration due to drop-out or loss to follow-up (~ 25%) a total sample size of 320 was targeted for recruitment.

### Data management and statistical analysis

Data were collected, double-entered and managed using REDCap electronic data capture tool hosted at Universitas Padjadjaran. SPSS version 28.0 was used for all analyses (IBM Corp, Armonk, NY). Primary analyses were undertaken as intention to treat. Pregnant women who experienced preeclampsia, eclampsia, still birth or multiple births or whose infants died, were born with congenital anomalies, or were a preterm birth, were excluded from the statistical analysis involving birth outcomes. The unit of randomization was the village, and the unit of analysis was the individual mother–infant dyad.

The primary and secondary growth outcomes, including birth length (cm) and birth weight (gr), and expressed as Z-scores, as well as attained length (cm), LAZ-score, and length velocity from birth to 5 mos post-partum (cm/mos) were compared between randomized groups using a general linear model which included village as a random effect and intervention as a fixed effect. Additional adjusted analyses were conducted, with covariates incorporated into the general linear model as appropriate. Maternal height and infant sex were included as covariates for birth outcomes. For growth outcomes at five months, models were additionally adjusted for human milk intake (grams per day) at five months postpartum. Analyses of maternal hemoglobin outcomes were adjusted for hemoglobin concentration at baseline (7–13 weeks gestation). For human milk intake, breastfeeding status (exclusive vs. partial breastfeeding) was included as a covariate.

## Results

**Fig. 1:**
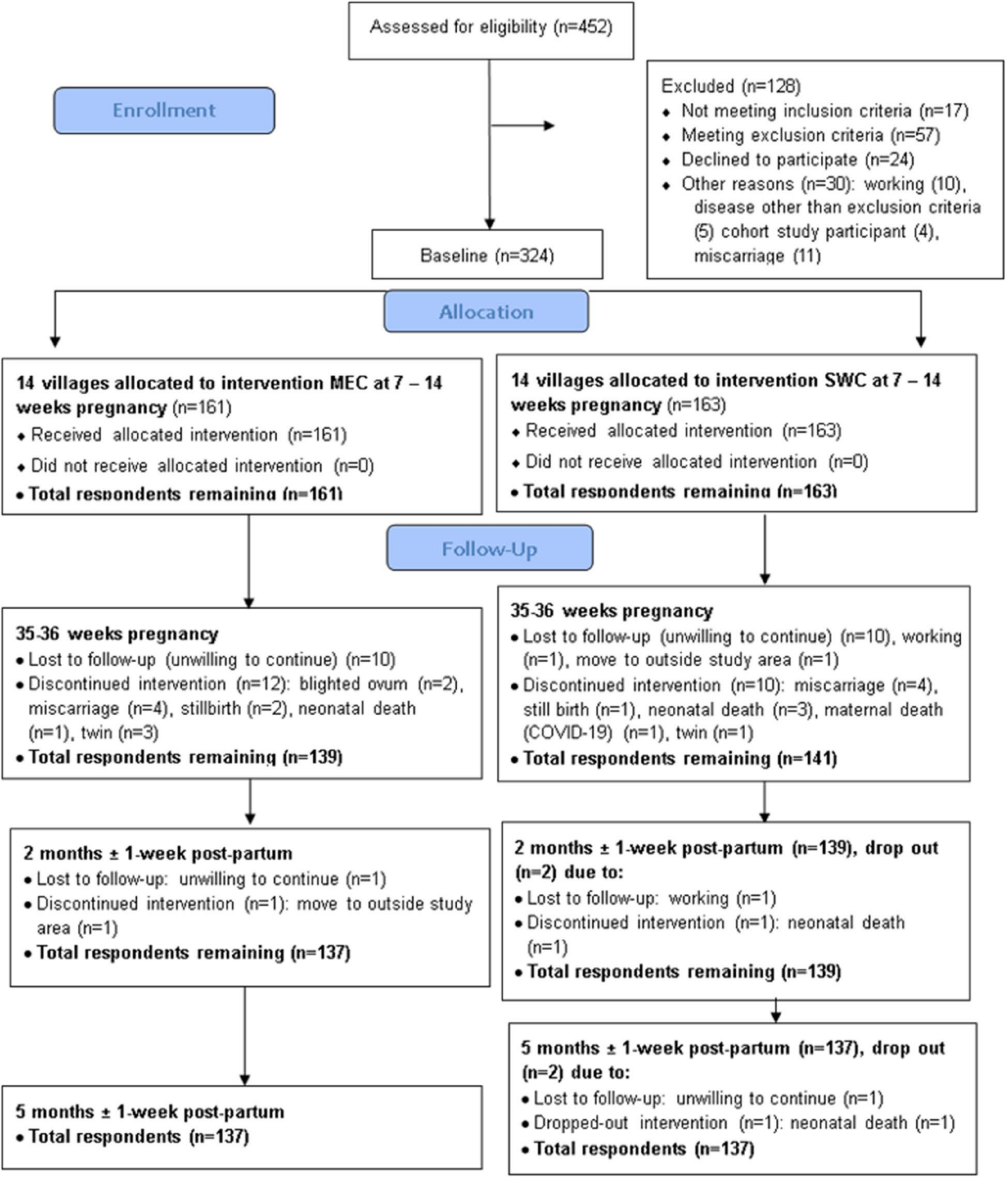
Consort flow diagram of Sustainable Intervention of Supplementation to Improve Kid's (SISTIK) Growth Study

A total of 452 women were screened for eligibility, of whom 324 were enrolled and randomized (Fig. 1). Recruitment was conducted from 12 November 2020 to 16 February 2022, with follow-up during home visits at 35–36 weeks gestation, 2 and 5 mos post-partum. Overall, refusal to participate (*n* = 20) was the major reason for discontinuing to 35–36 weeks gestation. An additional 22 women were excluded due to blighted ovum, miscarriage, stillbirth, neonatal/maternal death, and twin pregnancy (Fig. 1). From birth to 5 mos post-partum, 6 more respondents (mother-infant dyads) refused to continue (Fig. 1). There were no differences between the two groups in the characteristics and number of women discontinuing the trial or among those women completing versus those discontinuing the trial. The retention rate from enrolment to 5 months post-partum was high (i.e., ~ 85%). A total of 137 mother-infant dyads per group completed the study.

### Maternal and household characteristics at baseline

The trial arms were generally balanced at baseline, except for maternal age, although this difference was not considered clinically important (Table 2). Overall, about two thirds of the participants (69%) owned their own house, but only 36% had a monthly income that exceeded IDR 2,000,000 (~ USD 135). Few sources of drinking water and sanitary facilities were categorized as unimproved [38] and did not differ between the two groups.

**Table 2:** Baseline characteristics among women, by treatment group

Parameters	MEC (*n* = 161)		SWC(*n* = 163)		Total (*n* = 324)	
	**Data presented as mean** ± **SD or n (%)**					
Age (years), mean ± SD	26.1 ± 4.4*		27.2 ± 4.1*		26.7 ± 4.3	
Education, n (%)						
No schooling	2 (1%)		4 (3%)		6 (2%)	
Primary	35 (22%)		34 (21%)		69 (21%)	
Secondary	118 (73%)		116 (71%)		234 (72%)	
Diploma/University	6 (4%)		9 (6%)		15 (5%)	
Hb (g/dL), mean ± SD	12.2 ± 1.3		12.5 ± 1.3		12.3 ± 1.3	
Anemia, n (%)	27 (17%)		22 (13%)		49 (15%)	
Height						
Maternal height (cm), mean ± SD	151.6 ± 5.4		151.3 ± 5.3		151.5 ± 5.4	
Maternal height < 145 cm, n (%)	23 (14%)		23 (14%)		46 (14%)	
Body mass index (BMI), n (%)						
Normal (18.5–22.9 kg/m^2^)	54 (34%)		57 (35%)		111 (34%)	
Overweight (23.0–24.9 kg/m^2^)	31 (19%)		36 (22%)		67 (21%)	
Obese (> 25 kg/m^2^)	76 (47%)		70 (43%)		146 (45%)	
Parity, n (%)						
Primipara	43 (27%)		33 (20%)		76 (24%)	
Multipara	118 (73%)		130 (80%)		248 (77%)	
Economic parameter, n (%)						
Own house	105 (65%)		120 (74%)		225 (69%)	
Monthly income > IDR 2.000.000 (~ USD 135)	56 (35%)		61 (37%)		117 (36%)	
Wealth Index, n (%)						
Quintile 1 (lowest)	35 (22%)		31 (19%)		66 (20%)	
Quintile 2 (second)	39 (24%)		27 (17%)		66 (21%)	
Quintile 3 (middle)	30 (19%)		32 (20%)		62 (19%)	
Quintile 4 (fourth)	24 (15%)		41 (25%)		65 (20%)	
Quintile 5 (highest)	33 (20%)		32 (19%)		65 (20%)	
Household characteristics, n (%)						
Improved drinking water sources	152 (94%)		146 (90%)		298 (92%)	
Improved sanitation facilities	151 (94%)		157 (96%)		308 (95%)	

The *p*-value is obtained from the Chi-Square test for categorical variables and the Mann–Whitney test or independent t-test for continuous variables

^*^Significantly different at *p*-value < 0.05

There were no differences between groups for maternal height, BMI classification, and parity. The overall mean ± SD maternal height was 151.5 ± 5.4 cm; 14% in each group had height < 145 cm. Overall 46% were obese, 21% overweight, with the remainder within the normal weight range [32]. Note that a mid-upper arm circumference (MUAC) < 23.5 cm was an exclusion criteria. Mean ± SD baseline Hb concentrations tended to differ slightly between the two groups: 12.2 ± 1.3 versus 12.5 ± 1.3 gr/dL (p = 0.061).

### Maternal and infant characteristics after enrolment

Mean ± SD gestation at enrolment was 11.4 ± 1.9 weeks with the mean ± SD duration of the intervention being approximately 25.2 ± 6.7 weeks gestation in both groups. Overall mean ± SD weekly weight gain (0.34 ± 0.15 kg) was also comparable in the two groups. Pregnancy weight gain from enrolment to 34–36 weeks gestation did not differ between the two groups and exceeded the U.S. recommended range for 63% in MEC group and 60% in SWC group [33]. Similarly, weight loss during pregnancy did not differ (MEC, 3% vs. SWC, 0%).

Adherence to the intervention was significantly higher in the group consuming SWC compared to the MEC group (median 165 vs. 103 gr/week; *p* = 0.000, respectively), compared to the recommended 525 gr/week (75 gr/day). However, the number of vitamin and/or mineral supplements consumed did not differ between the two groups. Overall, approximately 81% of women consumed ≥ 90 tablets of either supplemental iron and/or folic acid and/or multi-micronutrients during pregnancy, with 69% consuming at least one calcium tablet.

The pattern of adherence during pregnancy continued post-partum, with median adherence for SWC higher than for MEC: 132 vs. 72 gr/week; *p* = 0.000. However, dietary supplement use was much lower during this period and comparable in the two groups: median = 23 tablets of iron and/or folic acid or multi-micronutrients over entire post-partum period.

Among households, the majority did not meet the water requirements of the Indonesian Ministry of Health at both the point of use for sanitation/hygiene [41] (MEC, 91% vs. SWC, 93%) and point of use for drinking [40] (75% vs. 52%), respectively, based on detection of *E coli* and other coliform bacteria*,* with no differences between the two groups. There were no differences in the number of male and female infants in each group.

### Growth outcomes

Preterm, LBW, and infants not breastfed (10% in each group) were excluded from all data analyses of infants at 5 mos post-partum. The intervention did not affect any of the primary or secondary growth outcomes in the unadjusted analysis (data not shown) and adjusted analysis. Primary growth outcomes were birth length (cm) expressed also as LAZ-score, as well as attained linear growth (cm) (also as LAZ-score) and length velocity (cm/mos) from birth to 5 mos (Table 3); birth weight (gr) and WAZ-score were secondary outcomes (Table 3).

**Table 3:** Growth outcomes among infants, by treatment group at birth and 5 mo of age; comparison of effect sizes and 95% CI

Variable	MEC	SWC	Mean difference	*p*-value
	**Mean (SE)**	**Mean (SE)**	**(95% CI)**	
**Birth**				
Length, cm^a^	48.3 (0.16)	48.4 (0.16)	−0.05 (−0.48, 0.39)	0.844
LAZ^b^	−0.58 (0.08)	−0.54 (0.09)	−0.04 (−0.27, 0.20)	0.752
Weight, gr^a^	2941 (35.7)	2986 (35.0)	−44.9 (−143.4, 53.7)	0.390
WAZ^b^	−0.62 (0.08)	−0.54 (0.08)	−0.08 (−0.30, 0.15)	0.488
**5 mo**^**c**^				
Length, cm	64.1 (0.21)	64.0 (0.22)	0.05 (−0.56, 0.66)	0.624
LAZ	−0.48 (0.10)	−0.50 (0.10)	0.02 (−0.26, 0.29)	0.768
Length velocity, cm/mo	3.04 (0.04)	3.00 (0.04)	0.04 (−0.07, 0.15)	0.667

All analysis were conducted using a general linear model which included village as a random effect and intervention as a fixed effect

*LAZ* length-for-age Z-score, *WAZ* weight-for-age Z-score

^a^Adjusted for maternal height and infant sex; all singleton livebirth infants were included (MEC (*n* = 139); SWC (*n* = 141))

^b^Adjusted for maternal height and infant sex; all singleton livebirth infants who were not preterm were included (MEC (*n* = 124); SWC (*n* = 120))

^c^Adjusted for maternal height, infant sex, and human milk intake; infants who were not LBW, not preterm, and were breastfed were included (MEC (*n *= 102); SWC (*n* = 92))

Furthermore, there were no differences between the two groups in the proportion of newborns with linear growth failure, underweight, wasting, IUGR, preterm birth, and LBW (Table 4). At 5 mos post-partum, there were no group differences in the proportions with linear growth failure and wasting. However, the proportion of underweight at 5 mos post-partum was higher in group receiving MEC (13%) than in SWC (3%) (*p*-value = 0.016).

**Table 4:** Binary growth outcomes among infants, by treatment group at birth and 5 mo of age; comparison of ORs with 95% CI

Variable	MEC	SWC	OR (95% CI)
**Birth**			
LAZ < −1^a^	39 (32%)	31 (26%)	0.76 (0.44, 1.33)
LAZ < −2^a^	4 (3%)	8 (7%)	2.14 (0.63, 7.31)
WAZ < −1^a^	38 (31%)	34 (28%)	0.90 (0.52, 1.55)
WAZ < −2^a^	5 (4%)	10 (8%)	2.16 (0.72, 6.53)
WLZ < −1^a^	22 (18%)	29 (25%)	1.50 (0.80, 2.79)
WLZ < −2^a^	2 (2%)	5 (4%)	2.68 (0.51, 14.08)
LBW (< 2500 gr)^b^	14 (10%)	19 (13%)	0.72 (0.35, 1.50)
Preterm (< 37 weeks)^b^	15 (11%)	21 (15%)	0.69 (0.34, 1.40)
IUGR (< 10th percentile)^b^	21 (16%)	24 (17%)	0.89 (0.47, 1.69)
**Variable**	**MEC (*n* = 102)**	**SWC (*n* = 92)**	**OR (95% CI)**
**5 mo**^**c**^			
LAZ < −1	29 (28%)	24 (26%)	0.89 (0.47, 1.67)
LAZ < −2	7 (7%)	4 (4%)	1.62 (0.18, 2.18)
WAZ < −1	38 (37%)	32 (35%)	0.90 (0.50, 1.62)
WAZ < −2	13 (13%)	3 (3%)	0.23 (0.06, 0.84)
WLZ < −1	31 (30%)	20 (22%)	0.64 (0.33, 1.22)
WLZ < −2	9 (9%)	5 (5%)	0.59 (0.19, 1.84)

*LAZ* length-for-age Z-score, *WAZ* weight-for-age Z-score, *WLZ* weight-for-length Z-score, *LBW* low birth weight, *IUGR* intra uterine growth retardation

^a^*p*-values and OR with corresponding 95% CIs comparing proportion of LAZ, WAZ, WLZ obtained from chi square or Fisher exact test to estimate parameters; all singleton livebirth infants who were not preterm were included (LAZ, WAZ: MEC (*n* = 124); SWC (*n* = 120), WLZ*: MEC (*n* = 123); SWC (*n* = 118))

^b^*p*-values and OR with corresponding 95% CIs comparing proportion of LBW, preterm, and IUGR obtained from chi square or Fisher exact test to estimate parameters; all singleton livebirth infants were included (LBW and preterm: MEC (*n* = 139); SWC (*n* = 141); IUGR**: MEC (*n* = 134); SWC (*n* = 139))

^c^*p*-values and OR with corresponding 95% CIs comparing proportion of LAZ, WAZ, WLZ obtained from chi square or Fisher exact test to estimate parameters; infants who were not LBW, preterm, and breastfed were included (MEC (*n* = 102); SWC (*n* = 92))

^*^Using the WHO Growth Chart (2016), the WLZ value does not appear when birth length < 45 cm, therefore a difference sample size

^**^Using the INTERGROWTH-21st (2022), the infants included born between 33 weeks + 0 days and 42 weeks + 6 days of gestational age, therefore a difference sample size

### Maternal hemoglobin (Hb), morbidity, and human milk intake

At 35–36 weeks gestation, there was no significant difference in mean ± SD maternal Hb concentrations (MEC group 12.11 ± 0.8 gr/dL vs. SWC group 12.22 ± 0.9 gr/dL), after adjusting for maternal Hb at enrolment (Table 5). The proportions with low Hb were similar for the two groups, when applying either the WHO threshold (Hb < 11 gr/dL) (i.e., 11.9% vs. 11.3%) [48] or the Hb < 3rd percentile threshold (i.e., 2% vs 0%) [47] for MEC and SWC groups, respectively. At 5 mo, there was also no significant difference between groups in maternal Hb levels.

**Table 5:** Maternal hemoglobin, morbidity, and human milk intake, by treatment group during pregnancy and 5 months post-partum; comparison of effect sizes and 95% CI

Variable	MEC	SWC	Mean difference	*p*-value
	**Mean (SE)**	**Mean (SE)**	**(95% CI)**	
Enrolment to birth				
Maternal hemoglobin 35–36 weeks^a^	12.11 (0.09)	12.22 (0.09)	−0.12 (−0.37, 0.14)	0.440
Maternal morbidity^b^				
Sick days, 3rd trimester	3.94 (0.48)	4.97 (0.49)	−1.03 (−2.37, 0.32)	0.281
Birth to 5 mo				
Maternal hemoglobin^c^	13.38 (0.08)	13.33 (0.08)	0.05 (−0.17, 0.27))	0.737
Maternal morbidity^b^				
Sick days, 2 mo	1.56 (0.39)	2.43 (0.39)	−0.87 (−1.96, 0.22)	0.096
Sick days, 5 mo	1.83 (0.40)	2.81 (0.40)	−0.98 (−2.09, 0.14)	0.214
Infant morbidity^b^				
Sick days, 2 mo	1.44 (0.31)	1.70 (0.32)	−0.25 (−1.14, 0.63)	0.489
Sick days, 5 mo	3.13 (0.43)	3.79 (0.43)	−0.66 (−1.84, 0.53)	0.284
Human milk intake (gr/day)^d^	767 (28)	830 (30)	−63.5 (−142, 15)	0.100

All analysis were conducted using a general linear model which included village as a random effect and intervention as a fixed effect

^a^Adjusted for maternal hemoglobin 7–13 weeks gestational age (GA) (MEC (*n* = 126); SWC (*n* = 133))

^b^The number of sick days was calculated from a record over 30 days at every visit (Sick days, 3rd trimester (MEC (*n* = 134); SWC (*n* = 133); 2 mos (MEC (*n* = 131); SWC (*n* = 128); Sick days, 5 mos (MEC (*n* = 126); SWC (*n* = 127))

^c^Adjusted for maternal hemoglobin 7–13 weeks and 35–36 weeks GA (MEC (*n* = 122); SWC (*n* = 125))

^d^Adjusted for breastfeeding practice (exclusive vs. partial breastfeeding) (MEC (*n* = 102); SWC (*n* = 92))

The low number of sick days for the women between 35 and 36 weeks gestation persisted for both the mothers and their infants at 2 and 5 mos post-partum, with no significant differences between the two groups.

There was no difference in breastfeeding status in the two groups with 58% of the infants exclusively breastfed in MEC vs. 64% in the SWC. Mean ± SD human milk intake measured over 14 days at 5 mos post-partum did not differ after adjusting for breast-feeding practice.

## Discussion

In this study we found no impact of the MEC on linear growth at birth or at 5 mos post-partum, despite the provision of the intervention from early pregnancy (i.e., 7–13 weeks GA) through 5 mos post-partum. Several factors may have contributed to the lack of a main intervention effect. First, the average intake of MEC (gr/day) by the intervention group during pregnancy was ~ 20% of the estimated 75 gr/day required to overcome the dietary micronutrient deficits reported earlier [4]. In addition, because the SWC contained fortified flour, their content of iron, zinc, thiamine, riboflavin, and folic acid approximated that of the MEC (Table 1). However, the bioavailability of iron and zinc was probably higher from MEC compared to SWC in view of the presence of animal source protein as one of its ingredients [54, 55]. Second, intake of micronutrient supplements during pregnancy was unexpectedly high with no difference between groups; overall 81% consumed ≥ 90 tablets of iron and/or folic acid or micronutrients compared to ~ 38% nationwide in Indonesia [56]. While IFA is provided through the national program, MMS and calcium are not routinely freely available in Indonesia and must be purchased, typically through local midwives. It is conceivable that promotion of use of all the dietary supplements (both IFA as well as other supplements) was intensified by the district midwives because of concerns about COVID-19. The cumulative effects of weekly home visits [57, 58], and maternal COVID concerns probably also played a role [59]. Adherence to the crackers was lower than expected. Qualitative findings pointed to taste fatigue, nausea—especially in early pregnancy—and the greater convenience of medicinal supplements. Some participants suggested that a less rigid schedule, such as intermittent consumption, might improve adherence by creating variation and renewing appetite. These insights highlight the value of participant feedback to enhance both acceptability and adherence when designing fortified food interventions. Finally, it is possible that an additional limiting factor may have been our exclusion of women with MUAC < 23.5 cm, preterm, and LBW infants.

It is of interest that in both groups the magnitude of linear growth failure at 5 mos post-partum was less compared to previous studies in the same Indonesian disadvantaged setting. For example, at 5 mos post-partum, the overall mean LAZ-score and stunting prevalence for these full term breastfed infants from resource-poor households were −0.49 (0.10) and 6% respectively, compared to −0.93 (0.85) and 11% in an earlier study in the identical setting and in which maternal height was comparable [60]. We speculate that such a reduction in linear growth failure was associated, at least in part, to the improved maternal and infant health care experienced by all participants during the trial, a trend reported previously in Indonesia [61], as well as the additional intake of micronutrients in both groups contributed by consumption of the crackers and micronutrient supplements.

We found no intervention effect on any of the secondary outcomes assessed, including birth weight, human milk volume, maternal and infant morbidity, and maternal hemoglobin concentration. However, maternal hemoglobin levels in both groups were higher than those reported in previous studies, even in those in which micronutrient supplements were reportedly consumed during pregnancy [62]. Overall, ~ 97% had a Hb within the normal range (i.e., >= 10th centile) at 35–36 weeks gestation [47]. Several factors may have contributed to the high Hb concentrations reported here, including the presence of micronutrient fortificants in the nationally fortified wheat flour used in both the SWC and the MEC, and the unexpected high compliance (81%) to iron and/or folic acid or multi-micronutrient supplements. Our implementation of health-seeking behaviors and nutritioneducation to all participants throughout the trial, together with improved health care through weekly home visits may also have played a role.

Changes in health seeking behaviors and improvements in health care may also be implicated in the low number of sick days during pregnancy and early infancy apparent in both groups, even though so few households had drinking water that met the criteria (i.e., no coliform bacteria) set by Indonesian Ministry (2010) [40]. Consequently, it is not surprising that illness did not modify the intervention effect on linear growth, unlike some earlier intervention studies [63, 64]. However, analysis of objective biomarkers of inflammation is needed to further explore this relationship.

The impact of sex (i.e., female vs. male) and maternal height on length at birth and later infancy is well documented in Indonesia [65–67], and has been confirmed earlier in this setting [24]. In our trial, neither sex (i.e., being female) nor maternal height modified the mean difference in the linear growth indices between the two groups at birth and 5 months post-partum. Furthermore, there were no differences between the two groups for the proportion of women with height < 145 cm, considered a proxy for generational influences on maternal height [68].

Some research suggests that optimum feeding practices during the postnatal period can mitigate the effects of poor fetal growth on linear growth faltering during early infancy. Through our DTM measurements at 5 mos post-partum, we confirmed 61% were exclusively breastfed (EBF) as recommended by WHO (2020) [46], and 28% partially breastfed at this time. Furthermore, the EBF infants received on average 798 gr/day at this time, an amount within the range reported for EBF infants aged < 6 mo worldwide [69, 70]. Furthermore, in the group overall there was a positive correlation between intake of human milk (gr/day) (via DTM) and LAZ-score at 5 mos post-partum. However, whether the micronutrient quality of the human milk consumed by the infants is also implicated in this relationship is unknown. In an earlier study in this same setting, we observed some relations between micronutrient quality in human milk and maternal biomarkers at 5 mos [71]. Therefore, further investigation of the micronutrient intakes and status of these mother-infant dyads is urgently required.

### Strengths and limitations in this trial

We believe our trial has several strengths. The design was a cluster randomized controlled trial based on locally produced MEC in which both the participants and staff involved in collection of all the measurements were blind to group assignment. Moreover, our intervention trial had rigorous quality assurance in all data collection, a high retention rate (~ 85%), and a design which optimized exposure to micronutrients during early pregnancy in an effort to enhance the rate of fetal growth [72].

As a result of COVID-19 lockdowns, however, hospital personnel performed length and weight measurements on 78/280 (28%) newborns. Nevertheless, sensitivity analyses revealed no significant differences for the two sets of measurements, and hence do not bias our conclusions. However, adherence to the daily consumption of crackers was lower than expected, despite encouragement through weekly home visits. Our low adherence may have been attributed in part to the introduction of the crackers early in pregnancy when nausea and vomiting are common [73]. Although dietary supplement use preconceptionally was unknown, our data throughout the trial were very detailed. Health care workers checked weekly the daily maternal records on dietary supplement use and verified the supplement brand names during home visits. While maternal serum micronutrient biomarker data could have provided additional insight into nutritional status and the potential effects of the intervention, these data are not included in the present analysis. Biomarker data were collected during the study and will be explored in future analyses.

Our GA data were based on retrospective maternal recall of first day of the last menstrual period, with pregnancy confirmed by a urine test because it was not feasible to use ultrasonography. However, in a blind RCT, any errors would be expected to be the same in both groups [74]. Moreover, in a recent meta-analyses report, the effects of multi-micronutrient and iron/folic acid supplements on adverse birth outcomes were similar, irrespective of the GA assessment method used [75]. We categorized gestational weight gain as inadequate, recommended, or excessive based on the U.S recommendations [33], after applying the BMI classification for Asians [32] at 7–13 weeks GA rather than at pre-pregnancy. Although BMI was assessed during pregnancy, it was classified using standard WHO adult BMI categories, which are commonly used for non-pregnant populations. In fact, national estimates from the 2023 Indonesian Health Survey report that among women aged > 18 years, 15.3% are overweight and 31.2% are obese, which aligns with the relatively high prevalence observed in our study sample. We also recognize that IOM recommendations for gestational weight gain may not be appropriate for the Asian population.

Morbidity assessments here were based on self-reported maternal records. We also tested household drinking water for *E.coli* and other coliform bacteria [76, 77], neither of which were associated with linear growth failure in either group at 5 mos post-partum. Clearly, the assessment of objective biomarkers of inflammation and EED are needed and will be examined in a later report. Finally, the generalizability of our findings to the wider population of pregnant women in Indonesia (i.e., external validity) is unknown. Our trial was restricted to low resource households in which women with MUAC < 23.5 cm and/or a Hb < 7 gr/dL were excluded, both characteristics likely to increase risk of poor fetal and infant growth.

## Conclusions

The key findings of our study highlight the complexity of addressing linear growth in low-resource settings. Despite meticulous efforts, adherence to the MEC intervention was low and did not prevent impaired linear growth. However, when compared to that of an earlier study in the same setting, linear growth in both groups improved at 5 months post-partum. This trend may be associated with the exposure by all the participants to intensive education on nutrition, health, and WASH. Hence, provision of such a multi-sectorial approach during pregnancy and early post-partum to reduce stunting in Indonesia warrants further investigation. In addition, a longitudinal follow-up could provide valuable insights on possible benefits for the children post-intervention.

## Data Availability

The datasets used and/or analyzed during the current study are available from the corresponding author on reasonable request.

## Abbreviations

BMI: Body mass index
DTM: Dose-to-mother
EBF: Exclusively breastfed
EED: Environmental enteric dysfunction
GA: Gestational age
Hb: Hemoglobin
IUGR: Intrauterine growth retardation
LMP: Last menstrual period
MEC: Micronutrient-enriched crackers
MUAC: Mid-upper arm circumference
RA: Research assistant
RCT: Randomized controlled trial
SISTIK: Sustainable Intervention of Supplementation to Improve Kid's
SWC: Standard wheat crackers
TEM: Technical error of measurement
